# Potential Implications of Mammalian Transient Receptor Potential Melastatin 7 in the Pathophysiology of Myalgic Encephalomyelitis/Chronic Fatigue Syndrome: A Review

**DOI:** 10.3390/ijerph182010708

**Published:** 2021-10-12

**Authors:** Stanley Du Preez, Helene Cabanas, Donald Staines, Sonya Marshall-Gradisnik

**Affiliations:** 1 National Centre for Neuroimmunology and Emerging Diseases, Menzies Health Institute, Griffith University, Gold Coast 4215, Australia; d.staines@griffith.edu.au (D.S.); s.marshall-gradisnik@griffith.edu.au (S.M.-G.); 2 Consortium Health International for Myalgic Encephalomyelitis, Menzies Health Institute Queensland, Griffith University, Gold Coast 4215, Australia; h.cabanas@griffith.edu.au; 3 School of Pharmacy and Medical Sciences, Griffith University, Gold Coast 4215, Australia; 4 School of Medicine and Dentistry, Griffith University, Gold Coast 4215, Australia; 5 Institut de Recherche Saint Louis, Université de Paris, INSERM U944 and CNRS UMR 7212, Hôpital Saint Louis, APHP, 75010 Paris, France

**Keywords:** transient receptor potential melastatin, ion channel, myalgic encephalomyelitis/chronic fatigue syndrome

## Abstract

The transient receptor potential (TRP) superfamily of ion channels is involved in the molecular mechanisms that mediate neuroimmune interactions and activities. Recent advancements in neuroimmunology have identified a role for TRP cation channels in several neuroimmune disorders including amyotropic lateral sclerosis, multiple sclerosis, and myalgic encephalomyelitis/chronic fatigue syndrome (ME/CFS). ME/CFS is a debilitating disorder with an obscure aetiology, hence considerable examination of its pathobiology is warranted. Dysregulation of TRP melastatin (TRPM) subfamily members and calcium signalling processes are implicated in the neurological, immunological, cardiovascular, and metabolic impairments inherent in ME/CFS. In this review, we present TRPM7 as a potential candidate in the pathomechanism of ME/CFS, as TRPM7 is increasingly recognized as a key mediator of physiological and pathophysiological mechanisms affecting neurological, immunological, cardiovascular, and metabolic processes. A focused examination of the biochemistry of TRPM7, the role of this protein in the aforementioned systems, and the potential of TRPM7 as a molecular mechanism in the pathophysiology of ME/CFS will be discussed in this review. TRPM7 is a compelling candidate to examine in the pathobiology of ME/CFS as TRPM7 fulfils several key roles in multiple organ systems, and there is a paucity of literature reporting on its role in ME/CFS.

## 1. Introduction

Neuroimmune disorders are a heterogenous collection of pathologies that feature both neurological and immunological dysfunction [1,2]. The recognition of neuroimmune disorders has grown in recent years with the identification of bidirectional biochemical communication and functional interplay between neurological and immunological mechanisms. Exploration of the cellular and biochemical interactions between the nervous and immune systems has identified a number of molecular mediators including cytokines, neurotransmitters, hormones [3], and the transient receptor potential (TRP) superfamily of ion channels in neuroimmune mechanisms [1].

TRP ion channels are polymodal sensors that mediate cellular adaptations in response to external stimuli. A growing body of research acknowledges a role for TRP ion channels in the pathophysiology of illnesses affecting neurological and immunological function, including myalgic encephalomyelitis/chronic fatigue syndrome (ME/CFS). ME/CFS is a multisystemic encephalitic disorder featuring metabolic, immunological, cardiovascular, autonomic, and neurological dysregulation. Fatigue unalleviated by rest, neuroimmune exhaustion, and exacerbation of symptoms including pain, fatigue, and impaired cognition following exertion define ME/CFS [4].

Recently, the TRP melastatin (TRPM) family of ion channels, namely TRPM3, as well as TRPM2 to a lesser extent, and calcium (Ca^2+^) signalling dysregulation have been implicated in the pathophysiology of ME/CFS [5,6,7,8,9,10,11,12,13,14]. The chanzyme, TRPM7, is a close phylogenetic relative of TRPM3 and TRPM2, suggesting TRPM7 dysfunction may also feature in the pathophysiology of ME/CFS. A growing body of research identifies a role for TRPM7 in metabolic [15,16,17,18,19], immunological [20,21,22,23], neurological [24,25,26,27,28,29,30,31], and cardiovascular [32,33,34] function. Similarly, dysregulation of TRPM7 channel-kinase activity is increasingly implicated in diseases affecting these systems [35,36,37]. In this review, the relevant biochemical properties of TRPM7 for metabolic, immunological, neurological, and cardiovascular physiology and pathophysiology will be outlined. Finally, their potential significance in the pathomechanism of ME/CFS will be discussed.

### 1.1. Biochemical Properties of Transient Receptor Potential Melastatin 7

The *TRPM7* gene and characteristics of the transcribed protein are highly conserved across vertebrate species [38]. The primary structure of transcribed TRPM7 includes six transmembrane domains (S1–S6), a pore-forming re-entrant loop located between S5–S6, and intracellular *C*- and *N*-termini, which are fundamental motifs present in all TRP ion channels, including TRPM2 and TRPM3 [39,40]. TRPM7 uniquely incorporates a constitutively active divalent cation channel domain fused to an α-type serine/threonine (S/T) protein kinase moiety [41]. These structural features enable TRPM7 to exhibit a dual function as a mechanism to enable entry of divalent cations and perform phosphorylation events. TRPM7 forms both pure TRPM7 homotetramers and heterotetramers with TRPM6 [42,43,44]. Moreover, knockdown of TRPM7 in primary cortical neurons also coincided with downregulation of TRPM2, suggesting that TRPM2 and TRPM7 monomers may be co-expressed in the central nervous system (CNS) [45]. Functionally, both TRPM2 and TRPM7 activity are modulated by reactive oxygen species (ROS), further suggesting a crosstalk between these two proteins [37].

### 1.2. Principal Regulators of Transient Receptor Potential Melastatin 7 Channel-Kinase Function

The TRPM7 ion channel displays a key role in magnesium (Mg^2+^), zinc (Zn^2+^), and Ca^2+^ entry [46,47]. TRPM7 ionic currents are principally regulated by Mg^2+^ and Mg^2+^-complexed adenosine triphosphate (ATP) (Mg·ATP) [46,48]. Mg^2+^-complexed adenosine diphosphate, however, demonstrates less potent inhibition of TRPM7 compared with Mg·ATP [16] and Mg^2+^-complexed adenosine monophosphate (AMP) fails to inhibit TRPM7 currents, suggesting a protective effect opposing TRPM7 activation during fluctuating energy states [16].

In support of this, mitochondrial inner membrane potentials and ATP synthase activity are also shown to modulate TRPM7 ionic currents [17]. By contrast, the TRPM7 kinase function is highly dependent on Mg^2+^, whereby phosphorylation of target S/T residues occurs proportional to Mg^2+^ availability [49]. Conversely, Ca^2+^ offers no modulatory effect on TRPM7 ion channel or kinase activity, and instead imparts a regulatory role via intermediates of Ca^2+^ signalling mechanisms, which principally includes the phospholipase C (PLC) pathway [15].

### 1.3. Transient Receptor Potential Melastatin 7 in Calcium- and Kinase-Signalling

Activation of PLC hydrolyses phosphatidylinositol-4,5-bisphosphate (PIP_2_) and promotes endoplasmic reticulum (ER) Ca^2+^ store release and activation of the mitogen-activated protein kinase (MAPK) pathway [50]. Current data indicates that transient depletion of PIP_2_ from PLC activation may potentiate TRPM7 ionic currents [51,52,53,54,55], and exhaustion of PIP_2_ reserves beyond an unknown limit renders TRPM7 inactive [56,57]. The TRPM7 kinase in turn increases phosphorylation PLC under conditions of low intracellular Mg^2+^ levels and disrupts G-protein-coupled receptor-mediated Ca^2+^ release when intracellular ATP levels are insufficient, thereby downregulating Ca^2+^ signalling [58,59].

Downstream stimulation of ER Ca^2+^ store release simultaneously engages a replenishing mechanism termed store-operated Ca^2+^ entry (SOCE). Depletion of ER Ca^2+^ content is detected by stromal interaction molecule (STIM), which interacts with the Ca^2+^ release-activated Ca^2+^ (CRAC) channels that augment Ca^2+^ signalling and initiate SOCE [60]. While not a store-operated Ca^2+^ channel, TRPM7 regulates SOCE [18]. The TRPM7 ion channel conveys Ca^2+^, while the kinase domain regulates STIM activation to promote CRAC channel activation and Ca^2+^ store replenishment [18]. Additionally, TRPM7 regulates intracellular Mg^2+^ homeostasis, which can further modulate Ca^2+^-dependent processes through natural antagonism of Ca^2+^ and activation of molecular mechanisms that regulate Ca^2+^ transport and availability [33,61,62].

The TRPM7 kinase also regulates the MAPK pathway via phosphoinositide 3-kinase (PI3K) in a Mg^2+^-dependent manner [58]. Silencing TRPM7 diminishes MAPK signalling [63] by reducing extracellular signal-regulated kinase (ERK) and c-Jun *N*-terminal kinase (JNK), but not p38 phosphorylation [64]. Correspondingly, overexpression of TRPM7 enhances both p38 and JNK activity via oxidative and nitrosative stress-dependent mechanisms [65].

Additional substrates regulated by TRPM7 for adaptive responses to stressors include nuclear factor kappa-light-chain-enhancer of activated B cells (NFκB), nuclear factor of activated T cells (NFAT), and transforming growth factor-beta (TGF-β) via phosphorylation of calpain [34]. TRPM7 also enhances AMP-activated protein kinase (AMPK) activation, which is a key regulator of cellular metabolism and glucose uptake [19]. Ca^2+^ influx through TRPM7 activates AMPK in neuronal cells [66,67], and upregulation of TRPM7 expression increases AMPK activity by 44% [65]. These signalling mechanisms regulated by TRPM7 are crucial to numerous physiological and pathophysiological processes.

## 2. Established Roles of Transient Receptor Potential Melastatin 7 in Physiology and Pathophysiology

### 2.1. Transient Receptor Potential Melastatin 7 Is Essential for Immune Function

TRPM7 is highly expressed by immune cells [68] and is integral to several immunological mechanisms [22]. In mast cells, the TRPM7 kinase participates in G-protein-regulated degranulation [69]. TRPM7-kinase modulates the sensitivity of degranulation to intracellular Ca^2+^ and extracellular Mg^2+^ by influencing the mobility and histamine content of granules [21].

TRPM7 may also have crucial roles in lymphocyte function, as B and T cell receptor (TCR) ligation triggers ER Ca^2+^ store release and nuclear translocation of NFAT, resulting in enhanced transcription of genes requisite for lymphocyte proliferation [20]. Importantly, homozygous kinase-deficient TRPM7 (*Trpm7^R/R^*) CD4^+^ T cells display diminished Ca^2+^ signalling following TCR stimulation [70,71]. Furthermore, T cells and B cells lacking TRPM7 have impaired SOCE [18,71], and produced size, growth, and proliferation defects [18]. Only overexpression of TRPM7 [18], extracellular supplementation with supraphysiological levels of Mg^2+^ (10 mM) [18,72], or sustained PI3K signalling [72] were able to reinstate the growth and proliferation of TRPM7-deficient B cells in regular media. These findings therefore highlight the role of TRPM7 and PI3K signalling in the regulation of lymphocyte growth.

TRPM7 also directs actin dynamics in B cells [23], where Mg^2+^ influx through the TRPM7 ion channel maintains myosin II-dependent cytoskeletal reorganisation and the TRPM7 kinase regulates non-muscle myosin IIA filament stability and actomyosin contractility [52]. Collectively, these mechanisms slow antigen internalisation and prolong B cell signalling [23]. Regulation of actin dynamics and Ca^2+^-dependent mechanisms by TRPM7 is also important in the CNS.

### 2.2. Transient Receptor Potential Melastatin 7 Regulates Neurological and Autonomic Processes

TRPM7 regulates several neurological processes including neuronal signalling and survival, synaptic transmission, and cognition [25,26,27,31]. TRPM7 is required for neuronal network differentiation and development, which are important in cognition. The growth cone vertices of developing neurites of primary mouse hippocampal neurons abundantly express TRPM7 [28]. Ca^2+^ influx through TRPM7 constrains axonal growth by regulating cytoskeletal organisation and growth cone extension [28].

In the presence of low Mg^2+^, the TRPM7 kinase also phosphorylates eukaryotic elongation factor 2 (eEF2) [28,73], which attenuates protein synthesis at maturing synapses. These processes ensure proper neuronal network development and the formation of robust synapses [28]. Moreover, TRPM7 kinase function and its regulation of actin dynamics are critically linked to synaptic plasticity [29]. Following TRPM7 suppression in the rat hippocampus or deletion in mouse glutamatergic neurons, synaptic plasticity is compromised [29]. Therefore, TRPM7 may have a central functional role in memory and cognition [30].

TRPM7 is also expressed on the surface of acetylcholine (ACh)-secreting synaptic vesicles of sympathetic neurons and in ACh-secreting small synaptic-like vesicles (SSLVs) [24]. TRPM7 expression levels show a positive correlation with cholinergic excitatory postsynaptic potential amplitude [74]. Genetic knock down of TRPM7 or disabling its ion channel function attenuates spontaneous and voltage-induced secretion of SSLVs [24]. Moreover, ACh-mediated vasorelaxation and downstream protein kinase B and nitric oxide synthase activation was impaired in heterozygous TRPM7 kinase deletion mutant (TRPM7^+/Δ*kinase*^) mice that received angiotensin-II [75]. These data suggest that TRPM7 regulates peripheral cholinergic vesicle mobilisation and, therefore, ACh-dependent processes including autonomic function. Importantly, autonomic nervous system dynamics influence cardiovascular function.

### 2.3. Transient Receptor Potential Melastatin 7 Is Implicated in Cardiovascular Disease

TRPM7 is implicated in an array of cardiac and vascular pathologies. TRPM7 messenger ribonucleic acid is downregulated in the left atria and left ventricles of patients with ischaemic cardiomyopathy [32]. TRPM7 expression levels showed a significant inverse relationship with left ventricular dysfunction, indicating changes in TRPM7 may affect cardiac function [32]. TRPM7-mediated Ca^2+^ influx, TGF-β pathway activation, and phosphorylation of ERK1/2 are believed to contribute to cardiac fibrosis and subsequent dysfunction of the myocardium [35,76,77,78]. In a separate study, the opposition of Ca^2+^-mediated pathological changes, including fibrosis, by TRPM7-dependent Mg^2+^ influx was proposed as the mechanism responsible for cardioprotection [60]. *Trpm7^+/^*^Δ*kinase*^ mice display cardiovascular inflammation and fibrosis [34]. These findings reflect dysregulation of macrophage activity and Mg^2+^-dependent processes. In support of this, intracellular Mg^2+^ supplementation relieved adverse repercussions in cardiac fibroblasts exposed to *Trpm7^+/^*^Δ*kinase*^ macrophages [34].

The effect of TRPM7 kinase deficiency may be explained by the dysregulation of macrophage activity and downstream TRPM7 kinase targets, namely calpain. Activation of the NFκB, NFAT, and TGF-β pathways by calpain predisposes to cardiac hypertrophy and fibrosis [34]. Mg^2+^ deficiency in vascular endothelial cells also correlates with increased ROS production and oxidative stress [33]. Supplementation with Mg^2+^ attenuates this effect. However, knockdown of TRPM7 prevents the protective effect of Mg^2+^ against oxidants occurring [33]. Similarly, the protective effects of TRPM7 and Mg^2+^ were attributed to the attenuation of Ca^2+^ ion channel activity and the antagonism of Ca^2+^-dependent processes that contribute to endothelial cell injury [32]. Considering the effect of TRPM7 in the regulation of these many mechanisms summarised in Table 1 may be of value in the pathophysiology of ME/CFS.

## 3. Implications of Transient Receptor Potential Melastatin 7 Dysregulation in Myalgic Encephalomyelitis/Chronic Fatigue Syndrome

### 3.1. Intracellular Signalling Dysregulation in Myalgic Encephalomyelitis/Chronic Fatigue Syndrome

TRPM7 participates in several physiological and pathophysiological processes at a systemic and molecular level, including Ca^2+^ signalling mechanisms. Impaired TRPM3 surface expression and function, reduced intracellular Ca^2+^ concentrations in natural killer (NK) cells and B lymphocytes [7], and dysregulated Ca^2+^ signalling are consistent findings in the NK cells of ME/CFS patients [7,8,9]. Although nine SNPs affecting TRPM3 were first identified by Marshall-Gradisnik et al., no SNPs affecting TRPM7 or TRPM2 were detected. However, increased expression of TRPM2 in the NK cells of ME/CFS patients was found in a subsequent investigation, which was suggested to reflect underlying Ca^2+^ signalling impairments [12].

Importantly, TRPM2 and TRPM7 may form heterotetramers [45]; hence, TRPM7 surface expression may also be altered in the NK cells of ME/CFS patients. The authors speculate that altered TRPM7 in ME/CFS may reflect or contribute to the altered cellular and biochemical features identified in ME/CFS patients thus far. Pathological differentiation of TRPM7 in ME/CFS may follow the dysregulation of metabolism or cellular mechanisms secondary to known TRPM3 and Ca^2+^ signalling impairments, epigenetic modulation of *TRPM7*, or post-translational modifications of the TRPM7 protein that influence the susceptibility of developing neuroimmune dysfunction. Importantly, TRPM7 is identified as a principal regulator of SOCE [18] and participates in Ca^2+^ influx as well as regulation of PLC [15,49,56,59]. Moreover, these mechanisms are also modulated by intracellular Mg^2+^ availability [33,58], which may be regulated by TRPM7 [33].

Early investigations revealed reduced intracellular Mg^2+^ concentrations in the erythrocytes of ME/CFS patients [79]. The same study reported that weekly intragluteal Mg^2+^ injections at a concentration of 1g/2mL for 6 weeks were beneficial and significantly improved patient symptom profiles [79]. However, other studies have either reported no Mg^2+^ deficit or beneficial effect of Mg^2+^ supplementation in ME/CFS [80,81,82]. Rationale for these equivocal results may be attributed to these later studies measuring extracellular Mg^2+^ levels, which are not representative of physiological Mg^2+^ levels, and the use of thresholds that fail to identify up to 50% of individuals with physiological Mg^2+^ deficiency [83,84]. Furthermore, only a single Mg^2+^ dose was administered [81], which is insufficient to manifest improvements [84]. Moreover, the functional status of ME/CFS patients was only reported on by the 6-week interventional study and not by the latter two studies, making the accurate comparison of outcomes difficult. Future investigations examining the role of Mg^2+^ in ME/CFS should therefore assess intracellular Mg^2+^ status and correlate this with the functional status of patients to determine whether cellular Mg^2+^ homeostasis, and by extension TRPM7 function, is impaired in ME/CFS.

Dysregulation of protein kinase gene expression and signalling pathways, such as perturbed MAPK signalling, has been characterised in the NK cells of ME/CFS patients [85,86]. Specifically, patient-derived NK cells show significantly decreased ERK1/2 phosphorylation as well as significantly increased MAPK kinase (MEK) 1/2 and p38 phosphorylation [86]. In support of these findings, other independent researchers using primary human skeletal muscle cell cultures from ME/CFS patients reported significantly impaired AMPK activation in response to electrical pulse stimulation [87].

Importantly, TRPM7 is known to regulate MAPK signalling via PI3K [58], and TRPM7 expression shows a positive correlation with AMPK activity [65,67]. Hence, TRPM7 may feature in the dysregulated signalling mechanisms reported in ME/CFS; however, this is yet to be investigated. Importantly, cellular mediators related to TRPM7 activity, including the MAPK family, AMPK, and PI3K, are key mediators of the adaptive responses to exercise [88].

### 3.2. Maladaptive Responses to Exercise in Myalgic Encephalomyelitis/Chronic Fatigue Syndrome

Decreased ATP production and increased lactate levels have been identified in ME/CFS compared with healthy controls [89], with greater differences apparent following repeat exercise tests in vivo [90]. These findings potentially indicate impaired oxidative phosphorylation in ME/CFS. Several mitochondrial processes including respiration are dependent on the availability of Mg^2+^, for example: cellular energy production, ATP synthase function, and ATP utilisation [91].

Moreover, Mg^2+^ regulates mitochondrial Ca^2+^ uptake as well as the metabolic and signalling effects of Ca^2+^ [91,92]. Additionally, depletion of intracellular Mg^2+^ inhibits glycolysis and glucose transportation [93]. Importantly, studies in ME/CFS patients have reported significantly impaired ATP production through glycolysis [94,95,96] and oxidative phosphorylation [94,95,96], and reduced ATP synthase activity [97]. As TRPM7 governs cellular Mg^2+^ homeostasis [33,58] and the activity of TRPM7 is intrinsically linked to the bioenergetic state of the cell [16,17,46,48], TRPM7 may therefore represent a molecular mechanism for compromised mitochondrial function in ME/CFS.

Increased serum glucose and decreased essential amino acid levels potentially provide evidence of dysregulated glycolysis, pyruvate dehydrogenase function, and oxidative phosphorylation in ME/CFS [94,97,98]. Essential amino acids may be used in ME/CFS as an alternative carbohydrate source for the tricarboxylic acid cycle to continue operating in the face of glucose hypometabolism [96,98]. Dysregulation of AMPK may explain increased serum glucose levels in patients as pharmacological activation of AMPK improves glucose uptake in skeletal muscle cells isolated from ME/CFS patients [87].

Symptoms of ME/CFS may be partially attributed to dysregulation of inflammatory, oxidative, nitrosative, and kinase signalling pathways, namely AMPK, that are regulated by TRPM7. Exercise increases ROS formation and normally promotes anti-inflammatory differentiation of the immune system through these signalling cascades that culminate in the down-regulation of NFκB, a relative increase in circulating anti-inflammatory cytokines, and decreased proliferation of microglial cells in the CNS [99,100,101]. In ME/CFS, however, exertion can instead exacerbate patient symptoms, including inflammation and other immunological manifestations.

### 3.3. Immunological Aberrations in Myalgic Encephalomyelitis/Chronic Fatigue Syndrome

Cytokine profiles in ME/CFS are disorderly and lack homogeneity for research and diagnostic purposes across patient cohorts [102] and may be related to increased MEK1/2 and p38 signalling [86]. Zn^2+^-gene interactions additionally modulate the production of cytokines including interleukin-6 and tumour necrosis factor-α (TNF-α) [103,104,105]. Moreover, Mg^2+^ deficiency increases inflammatory cytokine production [33]. Both intracellular Zn^2+^ and Mg^2+^ availability are regulated in part by TRPM7, hence this protein may be involved in the dysregulated cytokine production in ME/CFS.

A systematic review has established that NK cell dysfunction is the most consistent immunological feature in ME/CFS patients [6]. NK cell function relies on the successful mobilisation and polarisation of cytotoxic granules and subsequent exocytosis at the immune synapse with a target cell; mechanisms that are impaired in ME/CFS [6]. These processes are dependent on Ca^2+^ signalling [106,107,108,109], MAPK signalling [106,108,110,111,112], and cytoskeletal dynamics and vesicle fusion [107,113,114], all of which are regulated by TRPM7 in other cell types.

TRPM7 dysfunction in B lymphocytes compromises actin dynamics and B cell function [23], which may also occur in other immune cells such as NK cells. Importantly, the TRPM7 kinase regulates actomyosin remodelling, which indirectly modulates TRPM7 channel activity [115]. Dysfunction of the TRPM7 kinase may therefore impair cytoskeletal reorganisation and Ca^2+^ and Mg^2+^ influx through the TRPM7 ion channel domain.

Furthermore, TRPM7 regulates mast cell degranulation [69] and may have a comparable role in NK cell degranulation. Reduced granzyme concentrations have also been identified in the NK cells of ME/CFS patients [116]. This may be explained by impaired transcription, possibly due to reduced intracellular Mg^2+^ availability and diminished eukaryotic elongation factor activity from TRPM7 dysregulation.

Moreover, reduced intracellular Ca^2+^ stores in the NK cells of ME/CFS patients [7,8] may, in part, be explained by altered TRPM7 expression or function, which physiologically allows Ca^2+^ into cells and regulates SOCE via its ion channel and kinase domains, respectively. Hence, TRPM7 may also participate in NK cytotoxic processes in ME/CFS patients and requires further investigation. Some of the mechanisms regulated by TRPM7 in immune cells are also integral to several neurological processes.

### 3.4. Neurological Abnormalities in Myalgic Encephalomyelitis/Chronic Fatigue Syndrome

Cognitive problems are an additional neurological feature of ME/CFS. Patients frequently report brain fog, which is the subjective experience of slowed thinking, difficulty focusing, and forgetfulness [117,118,119]. Furthermore, neuroimaging studies in ME/CFS patients have identified hypoconnectivity in the brainstem and core neurocognitive networks [120,121].

ME/CFS patients additionally display an increased incidence of autonomic impairments [4]. Single nucleotide polymorphisms (SNPs) affecting ACh receptors as well as TRP ion channel members have been identified in ME/CFS patients, suggesting an association between ACh receptors and TRP family members in ME/CFS [122,123]. In support of this notion, TRPM3 activity is inhibited by G_q_-coupled muscarinic ACh receptor 1 activation [124], thereby connecting TRP ion channel activity with ACh receptor stimulation.

TRPM7 is highly expressed in the human CNS [125] and approximates TRPM3 as the predominant TRP ion channel expressed in the brain [126]. Moreover, TRPM7 is involved in learning, memory, and cognitive function, as well as regulating synaptic density in key areas including the hippocampus through the regulation of actin dynamics and protein transcription [28,29,30]. Therefore, TRPM7 dysregulation may partially explain some of the neurocognitive symptoms of ME/CFS.

Indeed, TRPM7 is implicated in regulating cholinergic vesicle release in peripheral neurons [24] and post-synaptic ACh-induced amplitudes also correlate with TRPM7 expression [74]. Hence, TRPM7 dysfunction may represent a molecular mechanism for autonomic dysfunction in ME/CFS. Autonomic dysfunction in ME/CFS also manifests in the cardiovascular system.

### 3.5. Cardiovascular Dysfunction in Myalgic Encephalomyelitis/Chronic Fatigue Syndrome

Cardiovascular compromise is a common complaint in ME/CFS. This may be due to a combination of autonomic dysfunction and maladaptive functional and structural cardiovascular responses to stressors. Evidence shows that ME/CFS patients die significantly earlier than the United States general population from cardiac failure (58.7 years vs. 83.1 years) [127]. Furthermore, ventricular dysfunction and increased arterial stiffness are noted in ME/CFS [104,127].

These factors contribute to the abnormal cardiovascular responses to mild orthostatic stressors observed in ME/CFS patients [128]. TRPM7 is known to support normal cardiac function and participates in the pathomechanisms that precipitate heart failure when dysregulated, namely cardiac fibrosis [32,34,35,76,77]. Furthermore, TRPM7 is a key regulator of vascular function and has a pathological role in vascular fibrosis [33,34,36]. The adverse effects of TRPM7 in the development of cardiovascular disease is attributed to pathological Ca^2+^ influx through the TRPM7 ion channel and dysregulation of targets downstream of the TRPM7 kinase [32,33,34,60]. Mg^2+^, however, is protective, as it competes with Ca^2+^ for binding sites at the TRPM7 ion channel pore and is essential for TRPM7 kinase function [49]. Although the role of Mg^2+^ in ME/CFS remains equivocal [82], dysregulation of TRPM7 and therefore cellular Mg^2+^ homeostasis with secondary Ca^2+^ signalling dysregulation in both the heart and vasculature may contribute to the development of cardiac failure in ME/CFS. The considerable involvement of TRPM7 in pathophysiological processes that are common to ME/CFS therefore suggest that this protein may represent a valuable therapeutic target to ameliorate disease burden in this disorder.

### 3.6. Transient Receptor Potential Ion Channels Melastatin 7 as a Potential Therapeutic Target for Mylagic Encephalomyelitis/Chronic Fatigue Syndrome

TRP ion channels are emerging as important therapeutic targets in several pathologies, including neuropathic pain, CNS dysfunction, and inherited pathologies [129,130,131]. Structure-based drug design, however, is limited by the incomplete resolution of TRP ion channel architecture [131]. The discovery of therapeutics that target TRP ion channels therefore currently relies on electrophysiological and fluorescence assays [131]. In the case of TRP-targeted drug discovery for ME/CFS, patch-clamp electrophysiological techniques have revealed a potential benefit of low-dose naltrexone (LDN, 3.0–5.0 mg/day), which antagonizes the µ opioid receptor and thus relieves TRPM3 inhibition [14].

Electrophysiological data indicate that naltrexone treatment restores TRPM3-like ionic currents in the NK cells of ME/CFS patients [10,14]. These findings are supported by clinical reports that demonstrate a positive response to LDN in ME/CFS. Polo et al. retrospectively analysed 218 ME/CFS patients and reported a positive treatment response in 73.9% of those receiving LDN therapy [132]. A three-patient case series also supports the use of LDN for ME/CFS [133]. Importantly, these clinical reports indicate that therapeutic targets and strategies additional to TRPM3 and LDN are required for ME/CFS, as 13.8% of patients in Polo et al.’s study were unresponsive to LDN, and a further 4.6% discontinued treatment because of adverse symptoms during the introductory phase [132]. TRPM7 may therefore be a valuable supplementary target alongside TRPM3 to relieve illness severity in ME/CFS.

Clinically relevant drugs that modulate TRPM7, however, are currently limited. Although naltrexone does not influence TRPM7 activity [134,135], other drugs used in clinical practice including fingolimod and aripiprazole appear to modulate TRPM7. Fingolimod is used in the treatment of relapsing-remitting multiple sclerosis (MS) and is an immunomodulatory agent that stimulates the sphingosine 1-phosphate receptor and inactivates the TRPM7 ion channel at µM concentrations [136,137]. Importantly, TRPM7 is implicated as a novel mediator of gliotic scar formation in the development of MS, as demonstrated by TRPM7 overexpression by reactive astrocytes in MS lesions [138].

Furthermore, fingolimod and the antipsychotic agent aripiprazole appear to dampen neuroinflammation. These drugs oppose TNF-α expression and the activation of the p38 MAPK pathway and SOCE, which is partially mediated through TRPM7 [139]. While the use of fingolimod in the treatment of ME/CFS is not reported in the literature, modulation of TRPM7 may be beneficial in the management of neuroimmune disorders. A retrospective study examining the use of low-dose aripiprazole (0.2–2.0 mg/day) in 101 ME/CFS patients found that 74% experienced symptomatic improvement [140]. Importantly, 12% were unresponsive to treatment, and 14% experienced an exacerbation of their symptoms during therapy [140], which reinforces the need to identify a repertoire of drugs and therapeutic targets for the clinical management of ME/CFS.

As the biological importance and clinical relevance of TRP ion channels, including TRPM7, become increasingly recognised, additional pharmacotherapeutics may be identified for the treatment of neuroimmune disorders including ME/CFS. Further characterisation of TRPM7 at the biochemical, cellular, and organismal level is therefore indicated to improve clinical outcomes for ME/CFS patients, as a sound theoretical rationale for this endeavour exists.

## 4. Conclusions

The TRPM7 channel-kinase is a ubiquitously expressed protein that participates in numerous cellular signalling networks involving Ca^2+^, Mg^2+^, and kinase cascades. Evidence of TRPM7 contributing to physiological and pathophysiological processes in immune, autonomic, cardiovascular, and metabolic processes is emerging. Dysregulation of these systems is noted in ME/CFS; however, there is a paucity of research demonstrating that TRPM7 may contribute to and similarly be affected by dysregulation of cellular and systemic processes inherent in ME/CFS. Therefore, both the ion channel and kinase domain of TRPM7 are compelling candidates to characterise in ME/CFS. Of particular interest is elucidating the role of TRPM7 in NK cell function in both healthy individuals and ME/CFS patients. Specifically, identifying the importance of TRPM7 in Ca^2+^ and kinase signalling cascades, as well as regulating intracellular Mg^2+^ homeostasis and metabolism, in NK cells may serve to facilitate the identification of additional diagnostic and treatment targets to relieve the burden of illness in ME/CFS.

## Figures and Tables

**Table 1:** Cellular, physiological, and pathophysiological roles of TRPM7. Abbreviations: ACh (acetylcholine), AMPK (adenosine monophosphate-activated protein kinase), CHO (Chinese hamster ovary), HEK (human embryonic kidney), MAPK (mitogen-activated protein kinase), Mg^2+^ (magnesium), PI3K (phosphoinositide 3-kinase), PLC (phospholipase C), TCR (T cell receptor), TRPM7 (transient receptor potential melastatin 7), TRPM7^+/Δ*kinase*^ (heterozygous TRPM7 kinase deletion mutant), TRPM7^R/R^ (homozygous kinase-deficient TRPM7).

Molecular Purpose of TRPM7	Model	Reference
Regulation of SOCE	In vitro, DT40 B lymphocyte cell line	[18]
	In vitro, TRPM7 kinase-dead mouse-derived splenocytes	[71]
Regulation of intracellular Mg^2+^ homeostasis	In vitro, vascular endothelium cell line	[33]
	In vivo and in vitro, TRPM7^+/Δkinase^ mice and TRPM7^+/Δkinase^ mouse-derived embryonic stem cells	[61]
Mg^2+^-dependent MAPK regulation via PI3K	In vitro, human breast cancer cell line	[63]
	In vitro, mouse primary cortical astrocytes	[64]
	In vitro, HEK-293 cell line	[65]
PLC regulation	In vitro, HEK-293 cell line	[15]
	In vitro, CHO and HEK-293 cell lines	[56]
	In vitro, DT40 B lymphocyte and HEK-293 cell lines	[58]
	In vitro, HEK-293 cell line	[59]
Calpain activation	In vivo, wild-type mice	[34]
	In vitro, HEK-293 cell line	[65]
AMPK activation	In vitro, HEK-293 cell line	[65]
	In vitro, human neuroblastoma cell line	[66,67]
Mast cell degranulation	In vitro, wild-type and TRPM7^+/Δkinase^ mice-derived peritoneal mast cells	[21]
TCR signal transduction	In vivo and in vitro, Trpm7^R/R^ mice and Trpm7^R/R^ mouse-derived gut lymphocytes	[70]
	In vitro, TRPM7 kinase-dead mouse-derived splenocytes	[71]
Regulation of lymphocyte size, growth, and proliferation	In vitro, DT40 B lymphocyte cell line	[18]
	In vitro, DT40 B lymphocyte cell line	[72]
Regulation of actin dynamics	In vitro, DT40 B lymphocyte cell line	[23]
	In vitro, mouse embryo-derived hippocampal neurons	[28]
	In vivo and in vitro, rat and mouse primary hippocampal cells and HEK-293 cell line	[29]
	In vitro, HEK-293 cell line	[52]
ACh signalling	In vitro, phaeochromocytoma cell line	[24]
	In vitro, rat-derived superior cervical ganglion cells	[74]
Cardiac and vascular fibrosis	In vivo, left ventricular biopsies of ischaemic cardiomyopathy patients	[32]
	In vivo and in vitro, Mg^2+^-deficient mice and vascular endothelial cells	[33]
	In vivo, wild-type mice	[34]
	In vivo, human cardiac tissue biopsies	[35]
	In vitro, Sprague Dawley rat-derived cardiac fibroblasts	[76]

## Data Availability

Not applicable.
